# Results of XEN45 Gel Stent Implantation in the Treatment of Primary Open-Angle Glaucoma Using 5, 10 or 20 *μ*g Mitomycin C: A Pilot Study

**DOI:** 10.1155/2024/3895054

**Published:** 2024-10-26

**Authors:** Felix F. Reichel, Vanessa Guggenberger, Hanna Faber, Jonas Neubauer, Bogomil Voykov

**Affiliations:** ^1^University Eye Hospital, Centre for Ophthalmology, Tübingen, Baden-Württemberg, Germany; ^2^Institute for Ophthalmic Research, Centre for Ophthalmology, Tübingen, Germany

**Keywords:** intraocular pressure, microinvasive glaucoma surgery, MIGS, mitomycin C, primary open-angle glaucoma, XEN45 gel stent

## Abstract

**Background:** No consensus has been reached on the adequate dose of mitomycin C (MMC) in XEN45 gel stent implantation. Lower doses have the potential to reduce MMC-linked side effects. This study aimed to evaluate treatment efficacy of ab interno XEN45 gel stent in primary open-angle glaucoma (POAG) with three different MMC doses.

**Methods:** This retrospective single-centre nonrandomised trail included 54 patients (56 eyes) who underwent XEN45 gel stent implantation for POAG with above-target intraocular pressure (IOP) under medical therapy. Eyes were grouped according to the received MMC dose: Group 1 (20 *μ*g; *n* = 21), Group 2 (10 *μ*g; *n* = 14) and Group 3 (5 *μ*g; *n* = 21). The primary endpoint was the mean IOP change in the three MMC dose groups after 6, 12 and 24 months. Secondary endpoints included the success rate defined as lowering of baseline IOP ≥ 20% and below a cut-off IOP set at three different levels: ≤ 18, ≤ 16 and ≤ 14 mmHg (Criteriums 1, 2 and 3), the mean number of ocular hypotensive medications and the frequency of needling procedures.

**Results:** After 24 months, the overall mean (standard error) IOP was significantly reduced from 24.7 (0.9) mmHg to 15.2 (0.7) mmHg (*p* < 0.0001). The average IOP change (standard error) in MMC dose groups 1, 2 and 3 was −8.6 (2) mmHg, −10.1 (2.1) mmHg and −10.4 (2.8) mmHg. Complete success (Criterium 1) was achieved in 50%, 62% and 43% of the eyes in groups 1, 2 and 3. No statistically significant difference was found within the first 24 months between the three MMC dose groups for IOP change, success rate, number of ocular hypotensive medications and the frequency of needling procedures.

**Conclusions:** XE45 was effective in all three dose groups. As the success rate did not significantly differ between the MMC doses, these results may support the use of the lowest dose.

**Trial Registration:** ClinicalTrials.gov identifier: 559/2016BO2

## 1. Introduction

Lowering intraocular pressure (IOP) is the main treatment for preventing optic nerve damage in primary open-angle glaucoma (POAG) [1]. While the trabeculectomy surgery has been the standard IOP-lowering intervention for many years, it may lead to several sight-threatening complications [2]. To reduce surgery-related adverse events, minimally invasive glaucoma surgeries (MIGS) have been developed as less-traumatic alternatives for mild-to-moderate glaucoma in patients who failed to achieve adequate control with conservative methods [3]. A variety of MIGS exist, using different pathways to improve aqueous humour drainage [3].

The XEN45 gel stent, a MIGS device, is a porcine hydrophilic gelatine tube with a length of 6 mm and an internal lumen diameter of 45 *μ*m which is usually implanted ab interno via a corneal incision through the sclera, draining to the subconjunctival space. The stent's dimensions are supposed to allow for a steady and consistent outflow of aqueous humour in order to prevent severe prolonged postoperative hypotony [4]. Nevertheless, the XEN45 gel stent implantation is a filtering surgical procedure, and as such, mitomycin C (MMC) has traditionally been used in order to reduce scaring of the filtering bleb [5–10].

While the efficacy and safety of the XEN45 gel stent with MMC application has been demonstrated in various types of glaucoma [11–14], there is currently no gold standard regarding the amount of MMC to be used. Although antimetabolites like MMC and 5-FU have been shown to reduce bleb failure, they have also been associated with prolonged hypotony, hypotony-related maculopathy, bleb leaks, bleb-related ocular infection, increased endothelial cell loss and fibrosis of the ciliary body [15–21]. Therefore, finding the minimally effective dose of MMC has the potential to reduce adverse side effects of the procedure. The objective of this study was to evaluate the treatment efficacy of the ab interno XEN45 gel stent in the treatment of POAG with three different MMC doses.

## 2. Materials and Methods

### 2.1. Study Design and Population

This retrospective single-centre nonrandomised study was performed at the University Eye Hospital in Tübingen, Germany. The influence of MMC dose was retrospectively assessed from data that were collected as part of this study on the clinical efficacy of the XEN45 gel stent. The study was performed in accordance with the tenets of the Declaration of Helsinki 1975 (2013 Revision) and was approved by the local independent ethics committee (IEC, project number 559/2016BO2). Informed consent was obtained from each patient prior to surgery.

The first XEN45 gel stent implantation at our clinic was performed in December 2015. All surgeries were performed by a single senior glaucoma surgeon (BV). In every case, MMC was used to reduce scarring of the filtering bleb. In Germany, the use of MMC is off-label, and therefore, application and dosage of MMC are at the discretion of the treating surgeon.

Initially, we followed the common expert recommendations for the dose of MMC, which was 20 *μ*g (0.1 mL of 0.2 mg/mL MMC). Since we noticed several avascular filtering blebs, we made a first MMC dose reduction to 10 *μ*g (0.05 mL of 0.2 mg/mL MMC) at 14 months after the first stent implantation at our centre, followed by an additional dose reduction to 5 *μ*g (0.025 mL of 0.2 mg/mL MMC) 3 months later. The 5 µg dose was then continued.

In order to investigate the effect of the dose reduction, all patients operated between September 2016 and July 2017 were analysed. This time period covered the 4 months before the first dose reduction from 20 µg to 10 *μ*g, the 3 months of the intermediate 10 µg dose and 4 months after the second dose reduction to 5 µg. This study design had the added benefit of including only patients operated 10 months after the first stent implantation in our centre, after well over 200 surgeries had been performed, which minimised learning curve effects.

All patients treated with XEN45 gel stent implantation for POAG insufficiently controlled by medication, defined by evidence of progressive optic nerve damage on optical coherence tomography (OCT) or visual fields and an IOP > 21 mmHg despite maximally tolerated medication, and aged ≥ 18 years and a minimum follow-up of six months after stent implantation were included into the study. Patients with any other type of glaucoma than POAG were excluded from the analysis. Patients where no reliable applanation tonometry was possible were also not included in the analysis.

From 151 eyes which received a XEN45 gel stent implantation during the study period, 56 eyes of 54 patients fulfilled the inclusion criteria.

The eyes were grouped according to the received MMC dose: Group 1 (20 *μ*g; *n* = 21), Group 2 (10 *μ*g; *n* = 14) and Group 3 (5 *μ*g; *n* = 21).

Outcomes assessed for each group included the IOP using Goldmann applanation tonometer, number of antiglaucoma medications, needling, time until needling and success rate.

As recommended by the World Glaucoma Association Guidelines, the success of the procedure was defined as a lowering of the baseline IOP ≥ 20% and below a cut-off IOP set at three different levels: ≤ 18 mmHg, ≤ 16 mmHg and ≤ 14 mmHg (Criteriums 1, 2 and 3) [22]. The success was complete if the target IOP was reached without antiglaucoma medication or qualified if antiglaucoma medication was necessary. A severe loss of visual acuity (loss of light perception) attributable to glaucoma or the requirement of further glaucoma surgery other than needling was considered as a failure of the procedure.

### 2.2. Surgical Procedure

Surgery was performed under topical anaesthesia with oxybuprocaine eye drops. The superonasal quadrant was marked at two points each at a distance of 3 mm from the limbus. MMC 0.2 mg/mL (0.1 mL, 0.05 mL or 0.025 mL in Groups 1, 2 and 3, respectively) was injected into the subconjunctival space penetrating the conjunctiva at 5 mm from the limbus at the 12 o'clock position. The use of a 1-mL syringe graded in 0.01-mL steps ensured accurate application of the desired amount of MMC. The bleb was then massaged over the area of the anticipated microstent insertion in the superonasal quadrant. By penetrating the conjunctiva at the 12h position, we aimed to avoid subconjunctival bleeding in the area of the intended filtering bleb, which is in accordance with the recommendations of the manufacturer. A main incision was then made inferotemporal 1 mm into the clear cornea using a 20G sideport knife. A smaller sideport incision was prepared at the 12 o'clock position. Lidocaine 1 mg/0.1 mL was given in the anterior chamber (AC), which was then filled with viscoelastic (HEALON®, Abbott Laboratories Inc., Abbott Park, Illinois, USA). The injector was placed in the main incision, and the needle was directed across the AC towards the superonasal quadrant. Proper positioning of the injector needle was confirmed by gonioscopy. The needle of the device was then used to penetrate the sclera aiming for the subconjunctival space 3.0 mm from the limbus. Then, the stent was carefully released, and the device was withdrawn. The proper placement of the microstent was confirmed. The viscoelastic was removed from the AC using balanced salt solution during which a filtering bleb formed. Intracameral Cefuroxime 1 mg/0.1 mL was given at the end of the procedure.

### 2.3. Pre- and Postoperative Management

Baseline IOP was assessed under antiglaucoma medication on the day before surgery. Treatment with antiglaucoma medication was stopped on the day of surgery. Moxifloxacin was started four times daily for one week on the first postoperative day. Additionally, unpreserved dexamethasone eye drops were tapered over five weeks, starting with five drops per day on the first postoperative day. Patients were seen on the first two postoperative days, with subsequent follow-up visits scheduled at two weeks and at 1, 3, 6, 9, 12, 18 and 24 months postsurgery. Visual acuity and IOP were measured at each follow-up visit.

In cases of inadequate IOP control (> 21 mmHg) associated with clinical signs of bleb scarring, a needling procedure was performed as the initial intervention, rather than prescribing antiglaucoma medication. Needling was performed by the same surgeon under topical anaesthesia using Conjuncain 0.4 mg/mL, without the application of MMC. A 30-gauge needle was inserted through the conjunctiva approximately 2 o'clock hours away from the bleb and advanced towards the gel stent. The fibrosis was then disrupted by a sweeping motion of the needle around the implant.

### 2.4. Statistical Analyses

The statistical analysis was performed with the JMP 16.2.0 software and R Studio, Version 2023.06.0 + 421. Data are presented as total numbers with percentages, mean values with standard deviation (SD), confidence interval (CI) or standard error (SE) where appropriate.

To compare the means between the three groups, one-way analysis of variance (*F*-test) and chi-squared tests were performed. The paired *t*-test was used to compare the IOP before and after XEN surgery.

Analysis of covariance (ANCOVA) was used to compare the IOP change between the MMC dose groups. The ANCOVA regression model included MMC concentration, age, sex, baseline IOP, number of glaucoma medications, history of glaucoma surgery and preoperative visual field mean defect as covariates.

Logistic regression analysis was done to test the association of factors with the success of the XEN surgery.

The level of statistical significance was set to *p* < 0.05.

## 3. Results

### 3.1. Baseline Values

From 151 eyes which received a XEN45 gel stent implantation during the study period, 56 eyes of 54 patients fulfilled the inclusion criteria. Groups 1, 2 and 3 included 21, 14 and 21 eyes, respectively. The baseline characteristics of the patients are shown in Table 1.

The mean IOP in the Groups 1, 2 and 3 was 24.2 ± 5.8 mmHg (mean ± SD), 24.2 ± 6.7 mmHg and 25.5 ± 8.2 mmHg, respectively. The mean number of antiglaucoma medications in the three groups was 3.6 ± 0.6 (Group 1), 3.0 ± 0.9 (Group 2) and 3.3 ± 0.8 (Group 3) at baseline. The three groups did not differ in respect of mean IOP at baseline (*F* (2, 53) = 0.23; *p*=0.79), antiglaucoma medication (*F* (2, 53) = 2.4; *p*=0.1), age (*F* (2, 53) = 0.15; *p*=0.86), sex distribution (*X*^2^ (2, *N* = 56) = 2.07; *p*=0.35), visual acuity (*F* (2,51) = 1.9, *p*=0.17), history of previous glaucoma surgery (*X*^2^ (2, *N* = 56) = 2.98; *p*=0.23), preoperative pseudophakia (*X*^2^ (2, *N* = 54) = 0.38; *p*=0.83) and field mean defect (*F*(2, 51) = 0.8, *p*=0.46).

### 3.2. IOP

**Figure 1:**
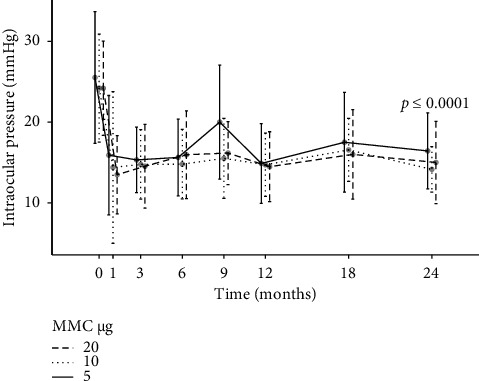
Intraocular pressure in Groups 1–3 before (Timepoint 0) and during 24 months after surgery. The different MMC doses are symbolised by different line types. Error bars show standard deviation (SD). Number of patients at different timepoints in months (Groups 1, 2, 3): 0/preoperative: *n* = 56 (21, 14, 21); 6: *n* = 56 (21, 14, 21); 12: *n* = 40 (17, 14, 16); 24: *n* = 33 (14, 14, 14).

The overall mean IOP reduction (SE) at 6, 12 and 24 months was 9.2 mmHg (1.2), 10.0 mmHg (1.2) and 9.7 mmHg (1.3), respectively (6, 12 and 24 months: all *p* < 0.0001; paired *t*-test) (Figure 1). The IOP reduction in percentage of baseline for the three MMC dose Groups 1, 2 and 3 was at 6 months: 31/34/33%; at 12 months: 34/36/39%; and at 24 months: 34/37/34%. There was no significant difference in IOP reduction between the three groups in the regression analysis adjusted for the presence or absence of previous surgeries, and the patients' age, sex, baseline IOP, antiglaucoma medication and visual field mean defect did not show any statistically significant difference in IOP reduction between the three dose groups (Table 2).

### 3.3. Antiglaucoma Medication

**Figure 2:**
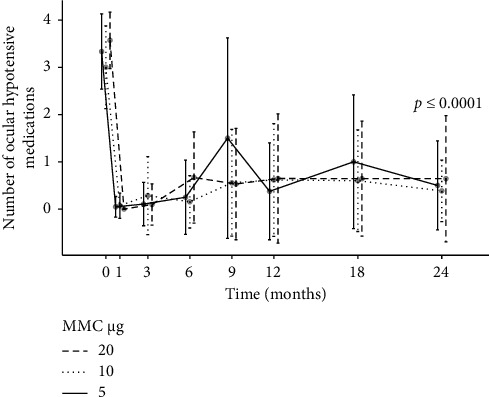
Number of ocular hypotensive medications in Groups 1–3 before and during 24 months after surgery. The different MMC doses are symbolised by different line types. Error bars show standard deviation (SD). Number of patients at different time points in months (Groups 1, 2 and 3): 0/preoperative: *n* = 56 (21, 14, 21); 6: *n* = 54 (21, 13, 20); 12: *n* = 40 (17, 13, 16); 24: *n* = 34 (14, 13, 14).

The mean number of antiglaucoma medication (CI) at 6, 12 and 24 months decreased statistically significant from 3.3 (3.1–3.5) to 0.4 (0.2–0.6) and 0.5 (0.2–0.9) and 0.5 (0.2–0.8) (all *p* < 0.0001, paired *t*-test) (see Figure 2).

After 6, 12 and 24 months, there was no significant difference in antiglaucoma medication between the three dose groups (6 months: *F* (2, 51) = 2.0; *p*=0.16; 12 months: *F* (2, 44) = 0.24; *p*=0.79; and 24 months: *F* (2, 38) = 0.22; *p*=0.8).

The different MMC doses are symbolised by different line types. Error bars show SD. The number of patients at different time points in months is given as follows (Groups 1, 2 and 3): 0/preoperative: *n* = 56 (21, 14, 21); 6: *n* = 54 (21, 13, 20); 12: *n* = 40 (17, 13, 16); and 24: *n* = 34 (14, 13, 14).

### 3.4. Needling

After 24 months, 59% of all patients had received needling. The patients that received needling, required a mean (CI) of 2.0 (1.7–2.4) needling procedures. No significant difference was found between the MMC dose groups and the number of needling procedures (*F* (2, 31) = 2.2, *p*=0.14).

**Figure 3:**
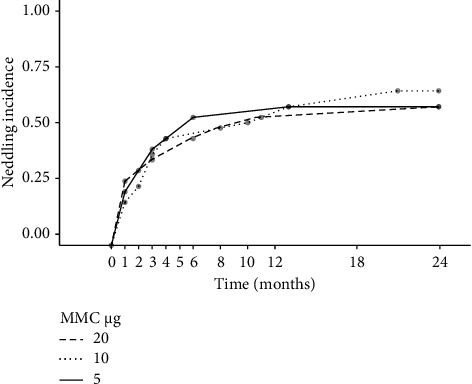
Needling prevalence in Groups 1–3 during the first 24 months after surgery. The different MMC doses are symbolised by different line types. A marker indicates the start and endpoints and whenever one or more needling procedures were performed.

The needling rate in Groups 1, 2 and 3 was 57% (12/21), 64% (9/14) and 57% (12/21), respectively. The first needling procedure was on average performed at 3.1 (0.6–5.7), 4.6 (0.6–8.3) and 2.0 (0.6–3.5) months after surgery in Groups 1, 2 and 3, respectively. The slight differences were found to be statistically not significant (*F* (2, 26) = 0.9, *p*=0.4), and no statistically significant difference was found between the overall probability of performing a needling procedure within the first 24 months and the MMC dose applied (*X*^2^ (2, *N* = 56) = 0.22; *p*=0.89) (Figure 3). Furthermore, no correlation between previous glaucoma surgery and the probability of performing a needling procedure was found (*X*^2^ (1, *N* = 56) = 0.90; *p*=0.34).

### 3.5. Success Rate

We evaluated the influence of MMC dose on treatment success according to the success criteria as defined in the method section at 12 and 24 months.

At 12 months, complete success (≤ 18 mmHg, Criterium 1) was reached in Groups 1, 2 and 3 in 71%, 57% and 69%. After 24 months, the complete success rate for Criterium 1 in Groups 1, 2 and 3 was 50%, 62% and 43%.

The success rate after 12 months was not significantly different between the three MMC dose groups neither for compete success Criteriums 1, 2 and 3 (*p*=0.71, 0.91, 0.30) nor for the qualified success Criteriums 1, 2 and 3 (*p*=0.23, 0.37, 0.49). Similar results were obtained at 24 months for complete success (*p*=0.62, 0.62, 0.66) and qualified success (*p*=0.54, 0.81, 0.91).

A logistic regression analysis to adjust for pseudophakia, previous glaucoma surgeries, the patients' age, sex, baseline IOP or visual field mean defect did not show a clinically significant effect of the three MMC dose groups on the complete success rates (Criterium 1) at 12 and 24 months (Table 3).

Preoperative pseudophakia was the only factor that was significantly associated with a higher complete success rate but only given the most strict Criterium 3 of ≤ 14 mmHg (*F*(1, 42) = 8.7, *p*=0.006). Thirteen of 27 pseudophakic eyes achieved this strict complete success, compared to only 1 of 14 phakic patients. We found no evidence for an interaction between MMC dose and lens status on achieving complete success for Criterium 3. Four out of 9 pseudophakic patients achieved this strict complete success with a dose of 20 *μ*g, while 5/9 achieved this with a dose of 10 *μ*g and 5/8 with a dose of 5 *μ*g.

### 3.6. Postoperative Complications

No patient exhibited any major complications such as endophthalmitis, malignant glaucoma or choroidal haemorrhages. Most patients developed postoperative hypotony, which is a common and well-known effect before the stent saturates. Asymptomatic transient hypotony, defined as IOP ≤ 6 mmHg, was observed in 57% (12/21), 78% (11/14) and 67% (13/21) of patients in Groups 1, 2 and 3, respectively (*p*=0.12). Symptomatic hypotony, defined by the requirement of treatment, occurred in three patients who needed intracameral Healon for choroidal detachment (two in Group 2 and one in Group 3). No patient presented with persistent hypotony.

## 4. Discussion

Comparing the outcomes of XEN45 gel stent implantation using 5, 10 and 20 µg of MMC, this nonrandomised retrospective study demonstrated no significant dose-dependent difference in IOP reduction or needling rate. The mean IOP reduction was 34%, 36% and 39% at 12 months and 34%, 37% and 34% at 24 months after surgery in Groups 1, 2 and 3, respectively. These reduction rates are comparable with the reported mean IOP reduction of the XEN45 gel stent, which ranges from 31% at 8.5 months to 35.6% at 12 months after surgery [5, 8, 10]. No statistically significant difference between the three MMC dose groups was found for the success rate within the first 24 months. To also analyse the effect of MMC on the needling rate, we compared the probability of performing a needling procedure and the time to first needling between the different MMC dose groups. Both parameters were not significantly associated with the amount of MMC applied.

These results do not reflect what could potentially be expected from in vitro studies which have demonstrated that the applied dose of MMC correlates with the concentration of MMC in the sclera and the amount of apoptosis induced in tenon's capsule fibroblasts [23, 24]. One could therefore expect a dose-dependent efficacy effect of MMC after XEN surgery. Yet, the majority of clinical studies on the efficacy of the XEN gelatine stent have each used a range of different MMC doses between 10 and 40 µg without analysing whether the success rate was associated with the MMC dose [5, 6, 8, 10, 25]. Only one recent retrospective study has reported no difference between 10 µg und 20 µg of MMC after a 12-month period [26].

Other studies that compared the effect of different MMC concentrations and exposure times for a classic trabeculectomy surgery did also not find a dose–effect relationship [27–32]. However, in trabeculectomy surgery, the comparison between different MMC doses is difficult as it usually involves MMC-soaked sponges that are applied for 1–5 min [33–41]. In the XEN45 gel stent surgery, the MMC is injected subconjunctivally which allows a better control over the exact amount of MMC applied and offers an improved comparability between different doses.

Because indications for performing a needling procedure are heterogeneous between centres, comparing the needling rates between studies is possible only with limitations. The reported needling rate after XEN45 gel stent implantation varies among 30.7% [5], 32.3% [8], 43.2% [6] and 45% [9], which is slightly lower than our results (57%–64%). This was likely caused by our aim to achieve complete success (no antiglaucoma medication) for the largest number of patients.

Although the MMC dose did not seem to have an effect on IOP reduction, pseudophakia was found to be an independent factor that was significantly associated with achieving the strictest success criterion after 24 months. Pseudophakic eyes achieved this criterion in 14 of 26 cases, compared to only one of 15 phakic eyes. The effect of pseudophakia on the outcome of the XEN45 gel stent procedure is disputed in the literature. Whereas some have reported greater success rates in pseudophakic eyes [10], others reported no difference in IOP reduction and number of medications between pseudophakic and phakic eyes after XEN45 gelatine stent implantation [42, 43].

Our study has several limitations to be mentioned. First, the patient cohorts were not randomised and analysis of the MMC effect was done retrospectively. Nevertheless, several measures were taken to minimise selection bias. The scheduling of the surgery was done by an independent team which was blinded to the study and the dose reduction was done at specific patient-independent timepoints. In between these timepoints, all patients at the surgical centre were treated with the same dose. The baseline characteristics of the three groups were analysed and did not significantly differ from each other. To further ensure homogeneity of the study population, only POAG patients were included. This affected the second limitation which is the limited number of patients included in the study with slightly uneven numbers of patients per group and a short minimum follow-up period of 6 months. Many non-POAG cases had to be excluded, and as the study was conducted at a tertiary centre, many patients were lost to follow-up. However, the majority of patients was followed up until 12 months and 24 months (88%, 49/56% and 77%, 43/56). Another limitation of the study results is that the patients were not washed out prior to surgery which is potential bias to the IOP-lowering effect of the procedure. We acknowledge that the study is underpowered for the detection of small differences in some of the variables and the number of patients included is too low to prove equivalence of the three MMC dose regimens.

## 5. Conclusion

In conclusion, our study found no difference for the MMC doses of 5 *μ*g, 10 *μ*g and 20 *μ*g in terms of IOP-lowering effect, needling rate and the time to first needling which may support the use of the lowest dose as equally efficient. Additional studies with larger patient cohorts are necessary to confirm these results to further investigate the minimally effective dose of MMC in XEN gelatine stent surgery.

## Figures and Tables

**Table 1:** Baseline clinical and ocular characteristics of included patients with primary open-angle glaucoma treated with XEN and MMC (*n* = 56 eyes of 54 patients).

Clinical factors	Total (*n* = 56)	20 *μ*g MMC (*n* = 21)	10 *μ*g MMC (*n* = 14)	5 *μ*g MMC (*n* = 21)	*p*
Age, years (mean [±SD])	67.5 (10.0)	68.1 (10.2)	66.3 (11.8)	67.8 (8.9)	0.86⁣^∗^
IOP, mmHg (mean [±SD])	24.7 (6.9)	24.2 (5.8)	24.2 (6.7)	25.5 (8.2)	0.79⁣^∗^
Number of medications (mean [±SD])	3.3 (0.8)	3.6 (0.6)	3 (0.8)	3.3 (0.8)	0.10⁣^∗^
Visual acuity, logMAR (mean [±SD])	0.36 (0.5)	0.52 (0.6)	0.33 (0.5)	0.23 (0.3)	0.17⁣^∗^
Visual field mean defect (mean [±SD])	11.2 (8.0)	12.7 (7.8)	11.9 (8.0)	9.5 (8.2)	0.46⁣^∗^
Sex, female (% [*n*])	35.7% (20)	23.8% (5)	42.9% (6)	42.9% (9)	0.35⁣^∗∗^
Preoperative pseudophakia (% [*n*])	66.7% (36)	66.7% (13)	71.4% (10)	61.9% (13)	0.83⁣^∗∗^
Combined surgery XEN + Phaco (% [*n*])	1.8% (1)	0% (0)	0% (0)	5% (1)	0.43⁣^∗∗^
Previous glaucoma surgery (% [*n*])	51.8% (29)	66.7% (14)	42.9% (6)	42.9% (9)	0.23⁣^∗∗^
Trabeculectomy (% [*n*])	30.4% (17)	38.1% (8)	28.6% (4)	23.8% (5)	0.60⁣^∗∗^
Cyclocryocoagulation (% [*n*])	8.9% (5)	19.0% (4)	0% (0)	4.8% (1)	0.10⁣^∗∗^
Cyclophotocoagulation (% [*n*])	32.1% (18)	47.6% (10)	21.4% (3)	23.8% (5)	0.16⁣^∗∗^
Other⁣^∗^	16.1% (9)	19.0% (4)	14.3% (2)	14.3% (3)	0.90⁣^∗∗^

⁣^∗^One-way ANOVA test.

⁣^∗∗^Chi-squared test.

**Table 2:** Comparison of mean change in intraocular pressure (IOP) after XEN implantation with 20 µg, 10 µg or 5 µg of mitomycin C (MMC).

	Total (*n* = 56)	20 *μ*g MMC (*n* = 21)	10 µg MMC (*n* = 14)	5 *μ*g MMC (*n* = 21)	*p* ^∗^
IOP change at 6 months, mmHg, mean (SE)	−9.2 (1.2)	−8.2 (1.6)	−9.4 (2.1)	−9.9 (2.3)	0.87
IOP change at 12 months, mmHg, mean (SE)	−10 (1.2)	−8.9 (1.8)	−9.5 (1.8)	−11.5 (2.6)	0.94
IOP change at 24 months, mmHg, mean (SE)	−9.7 (1.3)	−8.6 (2)	−10.1 (2.1)	−10.4 (2.8)	0.33

Abbreviation: SE = standard error.

⁣^∗^Analysis of covariance with the inclusion of MMC concentration, age, sex, baseline IOP, number of glaucoma medications, history of glaucoma surgery and preoperative visual field mean defect as covariates.

**Table 3:** Logistic regression analysis for complete success Criterium 1 (≤ 18 mmHg and ≥ 20% reduction of baseline IOP) at 12 months and 24 months after XEN surgery with three different mitomycin C (MMC) dose groups.

Factor	12 months		24 months	
	Odds ratio (CI)	*p*	Odds ratio (CI)	*p*
Age ≥ median (68 years)	1.7 (0.5–6.2)	0.42	0.85 (0.2–3.2)	0.81
Sex (M/F)	1.3 (0.3–5.3)	0.68	2.0 (0.5–8.5)	0.34
Baseline MD ≥ median (12 db)	0.6 (0.1–2.5)	0.46	1.0 (0.2–4.2)	1
Baseline IOP ≥ median (24 mmHg)	2.6 (0.7–10.3)	0.16	1.8 (0.4–7.8)	0.4
Previous glaucoma surgery	1.0 (0.2–4.8)	0.97	1.0 (0.2–5.6)	0.99
Pseudophakia	0.8 (0.1–4.1)	0.75	0.3 (0.1–2.0)	0.25
MMC dose Group 1/Group 2	0.5 (0.1–2.4)	0.38	1.4 (0.3–7.8)	0.67
MMC dose Group 1/Group 3	0.7 (0.1–3.6)	0.69	0.7 (0.1–3.5)	0.63
MMC dose Group 2/Group 3	1.4 (0.3–7.1)	0.64	0.7 (0.1–3.5)	0.37

Abbreviation: CI = 95% confidence interval.

## Data Availability

The retrospective data used to support the findings of this study are included within the article.
